# Exogenous Flupirtine as Potential Treatment for CLN3 Disease

**DOI:** 10.3390/cells9081872

**Published:** 2020-08-11

**Authors:** Katia Maalouf, Joelle Makoukji, Sara Saab, Nadine J. Makhoul, Angelica V. Carmona, Nihar Kinarivala, Noël Ghanem, Paul C. Trippier, Rose-Mary Boustany

**Affiliations:** 1 Department of Biochemistry and Molecular Genetics, American University of Beirut Medical Center, Beirut 1107 2020, Lebanon; kg21@aub.edu.lb (K.M.); jm53@aub.edu.lb (J.M.); sas85@mail.aub.edu (S.S.); nm36@aub.edu.lb (N.J.M.); 2 Department of Pharmaceutical Sciences, College of Pharmacy, University of Nebraska Medical Center, Omaha, NE 68198-6120, USA; angelica.carmona@unmc.edu (A.V.C.); paul.trippier@unmc.edu (P.C.T.); 3 Department of Pharmaceutical Sciences, Jerry H. Hodge School of Pharmacy, Texas Tech University Health Sciences Center, Amarillo, TX 79106, USA; Nihar.kinarivala@gmail.com; 4 Department of Biology, American University of Beirut, Beirut 1107 2020, Lebanon; ng13@aub.edu.lb; 5 Fred & Pamela Buffett Cancer Center, University of Nebraska Medical Center, Omaha, NE 68198-6120, USA; 6 UNMC Center for Drug Discovery, University of Nebraska Medical Center, Omaha, NE 68198-6120, USA; 7 Neurogenetics Program, AUBMC Special Kids Clinic, Division of Pediatric Neurology, Department of Pediatrics and Adolescent Medicine, American University of Beirut Medical Center, Beirut 1107 2020, Lebanon; 8 Neurogenetics Program and Pediatric Neurology, Departments of Pediatrics, Adolescent Medicine and Biochemistry, American University of Beirut, P.O. Box 11-0236 Riad El Solh 1107 2020, Beirut 1107 2020, Lebanon

**Keywords:** sphingolipids, neurodegeneration, ceramide, CLN3 disease, *Cln3^Δex7/8^* mice, flupirtine, allyl carbamate derivative, apoptosis

## Abstract

CLN3 disease is a fatal neurodegenerative disorder affecting children. Hallmarks include brain atrophy, accelerated neuronal apoptosis, and ceramide elevation. Treatment regimens are supportive, highlighting the importance of novel, disease-modifying drugs. Flupirtine and its new allyl carbamate derivative (compound 6) confer neuroprotective effects in CLN3-deficient cells. This study lays the groundwork for investigating beneficial effects in *Cln3^Δex7/8^* mice. WT/*Cln3^Δex7/8^* mice received flupirtine/compound 6/vehicle for 14 weeks. Short-term effect of flupirtine or compound 6 was tested using a battery of behavioral testing. For flupirtine, gene expression profiles, astrogliosis, and neuronal cell counts were determined. Flupirtine improved neurobehavioral parameters in open field, pole climbing, and Morris water maze tests in *Cln3^Δex7/8^* mice. Several anti-apoptotic markers and ceramide synthesis/degradation enzymes expression was dysregulated in *Cln3^Δex7/8^* mice. Flupirtine reduced astrogliosis in hippocampus and motor cortex of male and female *Cln3^Δex7/8^* mice. Flupirtine increased neuronal cell counts in male mice. The newly synthesized compound 6 showed promising results in open field and pole climbing. In conclusion, flupirtine improved behavioral, neuropathological and biochemical parameters in *Cln3^Δex7/8^* mice, paving the way for potential therapies for CLN3 disease.

## 1. Introduction

The neuronal ceroid lipofuscinoses (NCLs) constitute a family of fatal pediatric neurodegenerative disorders that primarily affect the central nervous system (CNS) [1]. NCLs are atypical lysosomal storage disorders that manifest accumulation of lipopigments in the lysosomes of neurons and other cell types [2]. CLN3 disease arises due to mutations in the CLN3 gene and is the most common variant of the NCL group [3]. This neurological disease manifests at four to eight years of age with progressive visual deterioration, seizures, blindness, motor and cognitive decline, mental retardation, epilepsy and early death during the second or third decade of life [4]. Massive cortical neuronal cell loss due to neuronal apoptosis within the cortex [5], and neuronal loss in hippocampus and microglial activation in this region are documented [6]. Eighty five per cent of patients with CLN3 disease harbor a 1.02 kb deletion eliminating exons 7/8 and creating a truncated CLN3 protein [7,8].

CLN3 protein influences major cellular functions, including apoptosis and cell growth 9. Apoptosis is the mechanism of neuronal and photoreceptor cell loss in human brain from patients with CLN3 disease 3 [9]. Ceramide, a pro-apoptotic lipid second messenger, mediates anti-proliferative events of apoptosis, growth inhibition, cell differentiation, and senescence [10].

Ceramide levels are increased in CLN3-deficient cells and in brain of CLN3 patients [11]. Studies confirm that CLN3 protein expression modulates brain ceramide levels. Levels of lipids ceramide, SM, GalCer, GluCer, and globoside are elevated in human CLN3-deficient fibroblasts. Ceramide levels normalized following restoration of CLN3 function, but not following caspase inhibition by zVAD, a pan-inhibitor of caspases [8,11,12]. Overexpressing CLN3 protein results in a drop in ceramide levels [13]. Increased ceramide levels and neuronal cell loss are evident in brain sections from post-mortem CLN3 disease patients and in brains and sera of *Cln3^Δex7/8^* mice [14]. Treatment regimens for CLN3 disease are largely supportive, not curative, and do not target the underlying causes of the disease.

Flupirtine is a centrally acting non-opioid drug previously widely used in clinics as an analgesic [15,16]. Flupirtine maleate is the salt of this drug, henceforth, referred to as just flupirtine. It is neuroprotective, has muscle relaxant and anticonvulsant properties [17] and suppresses neuronal hyper-excitability [18]. Flupirtine protects photoreceptor and neuronal cells from apoptosis induced by various insults [13]. There is evidence suggesting that flupirtine reduces brain injury, induces remodeling of brain tissue, and diminishes cognitive impairment in in vivo animal models of ischemic stroke [15]. Flupirtine protects lymphoblasts, differentiated human post-mitotic hNT neurons and PC12 neuronal precursor cells from apoptosis induced by etoposide [13,19]. A newly synthesized allyl carbamate derivative of flupirtine (compound 6) has shown potential neuroprotective effects in vitro, as one of nine flupirtine aromatic carbamate derivative [19,20,21]. Compound 6 imparted a 150% increase in Bcl-2/Bax ratio in vitro which is protective [19]. Flupirtine and its allyl carbamate derivative (compound 6) increased cell viability in human CLN3 patient-derived lymphoblasts and in neuronal precursor PC12 cells transfected with siRNA directed against CLN3, exhibiting significant anti-apoptotic and neuroprotective effects [19].

This study tests, in vivo, oral supplementation of flupirtine for a period of 14 weeks in *Cln3^Δex7/8^* knock-in mice. Outcomes of efficacy include improved behavioral measures, altered gene expression profiles, decreased glial immunoreactivity, and increased neuronal cell numbers in specific brain regions. Supplementation of compound 6 assessed effectiveness in several parameters, as a first step in also developing it as potential treatment for CLN3 disease.

## 2. Materials and Methods

### 2.1. Animals

This mouse work was conducted in accordance with an approved American University of Beirut (AUB) Institutional Animal Care and Use Committee (IACUC) protocol (IACUC approval #18-08-496). Animal testing was carried out at the AUB Animal Care Facility where animals were housed. C57BL/6J (JAX stock number: 000664) and homozygous *Cln3^Δex7/8^* (JAX stock number: 029471) mice were obtained from the Jackson laboratory, kept in a 12-h light/dark cycle (lights onset at 6:00 am) and supplied with access to food and water ad libitum. Room temperature was maintained between 18–26 °C, and relative humidity between 30–70%. Mice were housed in groups of 3–4/cage. All efforts to minimize number of animals and animal suffering were applied. Mice were monitored for weekly weights, basic behavior and general health throughout the study, and were bred in-house.

### 2.2. Flupirtine and Compound 6 Treatment

**Figure 1:**
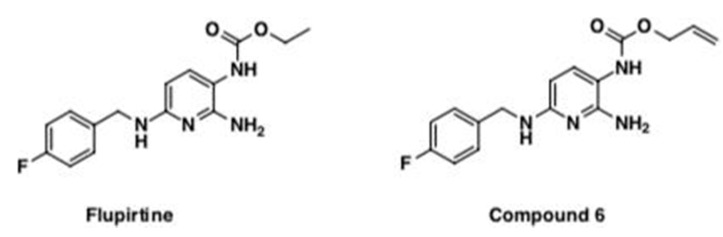
Chemical structure of flupirtine and its allyl carbamate derivative, compound 6.

Flupirtine and compound 6 were dissolved in vehicle (0.5% di-methyl sulphoxide (DMSO) in 10% phosphate-buffered saline (PBS)), at a dose of 30 mg/kg body weight for a period of 14 weeks starting at 4 weeks of age. Vehicle treatment consisted of 0.5% DMSO in 10% PBS. Mice were treated ‘per os’ by drinking water with a consistent supply in a volume of ≈8 mL/day/mouse. Both drugs were synthesized by Dr. Paul Trippier at the department of pharmaceutical sciences in the School of Pharmacy at Texas Tech University Health Sciences Center (Figure 1). Mice were divided into five groups, consisting of 16 mice each (eight males and females) and consisted of C57BL/6J vehicle-treated WT mice, C57BL/6J compound 6-treated WT mice, vehicle-treated *Cln3^Δex7/8^* mice, flupirtine-treated *Cln3^Δex7/8^* mice and compound 6-treated *Cln3^Δex7/8^* mice. Genotype was confirmed by PCR of DNA mouse blood.

### 2.3. Behavioral Studies

Mice were held in their cages in the behavioral room for testing, with lights dimmed for 60 min prior to onset of tests, for habituation. All behavioral assays were performed during the light cycle. Each test was performed at *n,* the same time of the day and within the same hour, when possible, to minimize variability between cohorts. Comparison between groups was carried out on males and females separately.

Open field: A mouse was placed on the periphery of a transparent Plexiglas cubic box (dimensions: 50 cm width, 50 cm length and 30 cm in height, with the center 16.4 cm^2^) so that locomotion would be apparent to the operator and exploratory behavior videotaped. Specific parameters were recorded by a top-mounted video-recorder using EthoVision software (Noldus Information Technology, Wageningen, The Netherlands) for each animal including total distance traveled, average speed, mobility duration, rearing and walling frequencies. Each mouse was allowed to move in the arena freely for 5 min. The box was wiped with 50% ethanol between each mouse to avoid olfactory cuing.

Pole climbing: Mice were habituated to the task in five trials/day for 1 day. On the next test day, five trial measures/mouse were performed by placing the mouse head upward on top of a rough- surfaced pole (1 cm in diameter and 60 cm in height) wrapped with tape to prevent slipping. The time for the mouse to completely turn its head down (t_turn_), time to reach the middle of the pole (t_1/2_), time it takes to descend and settle on the floor (t_total_), and time the mouse spent freezing during descent (t_stop_) were recorded. Each mouse had a maximum time of two minutes to climb down to avoid exhaustion.

Morris water maze (MWZ): The spatial learning abilities of mice were assessed in a MWM task. The apparatus consisted of a circular pool 100 cm diameter, filled up to 50 cm with water made opaque by addition of a small amount of non-toxic white paint and maintained at 21–22 °C. The pool is virtually subdivided into four quadrants using the software. A circular escape platform was placed in a fixed south-west (SW) location hidden 0.5 cm below the surface of the water, and five fixed-position geometric visual cues were kept in the brightly lit room throughout the period of testing. A digital camera was positioned above the center of the tank and linked to a tracking system (ANY-Maze behavioral tracking software, version 6.3, USA) in order to record escape latencies, path distance (m), percentage of thigmotaxis path and swim speed for each trial, together with time spent swimming in each of the four quadrants of the maze.

Mice (eight males and females from each treatment group) were given four consecutive days of acquisition training sessions that consisted of four trials per day. Throughout the course of this acquisition period the position of the hidden platform remained fixed (SW) and the entry point was varied from trial to trial, but the sequence remained fixed for all mice within that day. We used four different entry points (north, south, east, and west). The sequence of starting points was modified from one day to the other. Mice were given 60 s to find the platform, and if the mouse failed to locate the platform within this period, it was guided onto it. All mice were allowed to rest on the platform for a 30-s interval after each trial. At the end of the training block, mice were put in a drying cage and allowed to dry prior to being returned to their experimental cages. The last day of the test, the ‘probe trial’ was performed with the platform removed from the maze and the rodent released from the North entry point of the pool to find the previous location of the hidden platform.

### 2.4. Corticosterone Immunoassay Kit

Blood was collected from the inferior vena cava, left to clot, centrifuged and spun for 15 min at 10,000 rpm at 4 °C. Serum corticosterone levels were determined using DetectX^®^ Corticosterone Enzyme Immunoassay Kit (Cat. No K014-H5, Arbor Assays, Ann Arbor, MI, USA), according to manufacturer’s instructions. Five μL of standards or mouse serum samples were assayed in duplicate and run altogether on one single plate simultaneously. The absorbance was measured using TriStar2S microplate reader (Berthold Technologies, Bad Wildbad, Germany) at 450 nm.

### 2.5. RNA Extraction from Brain Tissue 

Mice were deeply anesthetized with a mixture of xylazine and ketamine (10 mg/kg and 100 mg/kg, respectively) and brains were rapidly dissected and “snap” frozen in liquid nitrogen to preserve RNA integrity, and stored at −80 °C. A total of 30 mg ground fresh brain tissue (from four males and females in each treatment group) were homogenized using a motorized rotor-stator homogenizer and RNA extracted using RNeasyPlus Mini Kits (Cat No. 74134, Qiagen, Germantown, MD, USA) according to manufacturer’s instructions. For assessing RNA quality, A260/A280 and A260/A230 ratios for RNA are analyzed with the ExperionTM Automated Electrophoresis System (BioRad, Hercules, CA, USA). RNA concentrations are determined by absorption at 260 nm wavelength with a ND-1000 spectrometer (Nanodrop Technologies LLC, Wilmington, DE, USA).

### 2.6. Quantitative Real-Time PCR (qRT-PCR)

Total RNA extracted from fresh brain tissues was reverse transcribed using RevertAid Reverse Transcriptase (Thermo Fisher Scientific, Waltham, MA, USA) with 2 µg of input RNA and random primers (Thermo Fisher Scientific, USA). qRT-PCR reactions were performed in 384-well plates using specific primers (Tm = 60 °C) (TIB MOLBIOL, Berlin, Germany) (Table 1) and iTaq SYBR Green Supermix (BioRad, Hercules, CA, USA) as a fluorescent detection dye, in CFX384TM Real-Time PCR (BioRad, USA), in a final volume of 10 µL. To characterize generated amplicons and to control contamination by unspecific by-products, melt curve analysis was applied. Results were normalized to β-actin or Gapdh mRNA level. All reactions were performed in duplicate, and results were calculated using the ∆∆Ct method.

### 2.7. Immunohistochemistry

For morphological and immunohistochemical sectioning, 4 mice/treatment group were deeply anesthetized with a mixture of xylazine and ketamine (10 mg/kg and 100 mg/kg, respectively) and fixed by cardiac puncture with 30 mL of 4% paraformaldehyde (PFA) in PBS. Brains were carefully isolated and fixed with 4% PFA solution (pH of 7.4) for 2 h at 4 °C, then cryoprotected in a solution of 20% sucrose overnight. The following day, brains were processed and frozen using embedding medium, Optimal Cutting Temperature (OCT) compound, for later tangential sectioning on glass slides. Brains were cut in coronal sections (20 μm) using a cryostat and sections stored at −20 °C for further analysis. Brain coronal cryosections were treated with PBS for 5 min twice, then incubated with PBST (0.1% Triton X-100 in PBS) for 10 min twice. Sections were incubated with blocking solution (PBST 0.1%-FBS 10%) for 1 h at room temperature (RT). They were then incubated with each of the primary antibodies: anti-GFAP (1:500, Abcam, Cambridge, UK, catalogue #ab7260) and anti-NeuN antibody (1/300, Abcam, Cambridge, UK, catalogue #ab104225) in antibody solution (PBST 0.1%-FBS 1%) overnight at 4 °C. After washing in PBST, slides were treated with Sudan black for 40 min, then washed with PBS three times. Brain cryosections were then incubated with biotinylated secondary antibody diluted in antibody solution at RT for 1 h. Samples were counterstained with 1:10,000 Hoechst (Sigma, St. Louis, MO, USA) and then mounted in Fluoromount (Sigma, St. Louis, MO, USA).

Signal quantification was assessed using Leica microscope software imaging. For microscopic imaging, three sections/mouse were selected. Primary motor cortex was viewed at 40× magnification with three to four photos/section for motor cortex layers (I–VI). Hippocampus images for GFAP were obtained at 40× magnification. Intensity quantification was depicted by the ratio of integrated density over total area of image using Image J software, version 1.52a. NeuN positive cells were quantified manually using Image J software. Number of NeuN positive cells divided by total area of image.

### 2.8. Statistical Analysis

Basic statistical analysis was conducted using GraphPad Prism 6 statistical package (GraphPad Software version 6.04, San Diego, CA, USA). Data was expressed as mean ± standard error of the mean (SEM). For two group comparisons, Student’s *t*-test was used with quantitative continuous variables. Comparisons between different groups were statistically tested with either one-way analysis of variants (ANOVA) or two-factorial ANOVA followed by Tukey’s post-hoc test for multiple group comparisons. All tests are two sided and a *p*-value < 0.05 is considered as statistically significant.

## 3. Results

### 3.1. Impact of Flupirtine on Motor Behavior of Homozygous Cln3^Δex7/8^ Mice

**Figure 2:**
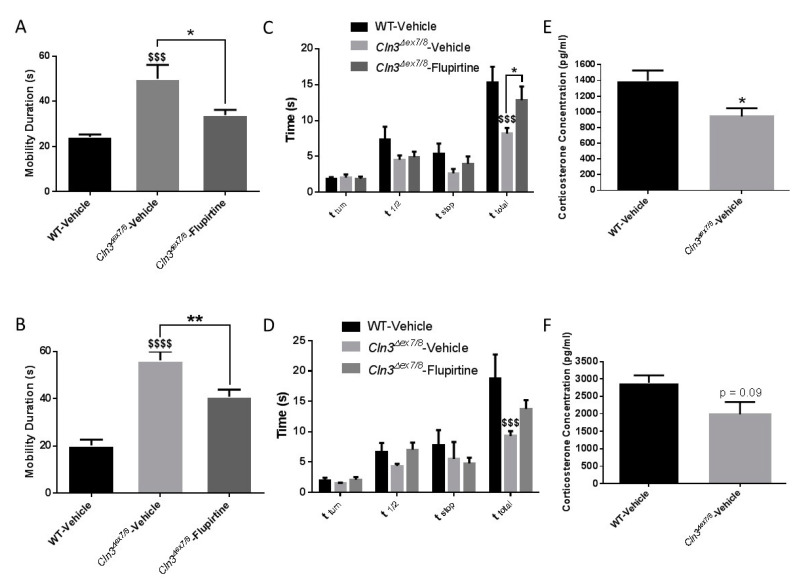
Impact of flupirtine on motor behavior of male and female *Cln3^Δex7/8^* mice. Open field behavioral parameter (mobility duration) in vehicle-treated WT, vehicle-treated *Cln3^Δex7/8^*, and flupirtine-treated *Cln3^Δex7/8^* (**A**) male and (**B**) female mice (*n* = 8 per group). Pole climbing test in vehicle-treated WT, vehicle-treated *Cln3^Δex7/8^*, and flupirtine-treated *Cln3^Δex7/8^* (**C**) males and (**D**) female mice (*n* = 8 per group). Time to turn (t_turn_), time needed to reach center of pole (t_1/2_), amount of time stopped (t_stop_), and total time to descend (t_total_) were recorded. All data are expressed as mean ± SEM. ^$^ *p*: compared to vehicle-treated WT mice; and * *p*: compared to vehicle-treated *Cln3^Δex7/8^* mice. * *p* < 0.05, ** *p* < 0.01, ^$$$^ *p* < 0.001, and ^$$$$^ *p* < 0.0001. Corticosterone levels in vehicle-treated WT and *Cln3^Δex7/8^* (**E**) male and (**F**) female mice (*n* = 8 per group). Data are expressed as mean ± SEM.

The open field behavioral test assesses novel environment exploration, general locomotor activity, and provides an initial screen for anxiety-related behavior in rodents. Open field behavioral testing showed that vehicle-treated *Cln3^Δex7/8^* mice exhibit increased spontaneous locomotor activity compared to vehicle-treated WT controls in both genders at 15 weeks of age. Vehicle-treated *Cln3^Δex7/8^* mice were significantly more mobile than their vehicle-treated WT littermates (Figure 2A,B). These results indicate that WT mice display increased anxiety-like behavior when put in the novel test environment compared with *Cln3^Δex7/8^* mice. Normally, rodents display distinct aversion to large, brightly lit, open and unknown environments. They have been phylogenetically conditioned to see these types of environments as dangerous [22]. To confirm whether *Cln3^Δex7/8^* mice have less anxiety than WT mice, we analyzed serum levels of the predominant murine glucocorticoid, corticosterone, in these animals. Male *Cln3^Δex7/8^* mice experienced significantly lower corticosterone levels than those measured in WT animals (Figure 2E). Similarly, female *Cln3^Δex7/8^* mice experienced lower corticosterone levels than WT animals, that was very close to significance (*p* = 0.09) (Figure 2F). These results confirm that WT mice have an enhanced physiologic response to stress, characterized by increased hypothalamic-pituitary-adrenal axis activity. Flupirtine treatment slowed down significantly the locomotor hyperactivity of male and female *Cln3^Δex7/8^* mice (Figure 2A,B).

The pole climbing test measures motor coordination, vertical orientation capability, and balance of mice. Vehicle-treated *Cln3^Δex7/8^* male and female mice descended the pole faster than vehicle-treated WT mice (Figure 2C,D), in line with the hyperactivity of *Cln3^Δex7/8^* mice observed in the open field experiment. In males, flupirtine supplementation had a significant impact on increasing and delaying the descent compared to vehicle-treated *Cln3^Δex7/8^* mice (Figure 2C). In females, flupirtine supplementation also trended to increase and delay the descent compared to vehicle-treated *Cln3^Δex7/8^* mice, however it did not reach significance (Figure 2D).

### 3.2. Impact of Flupirtine on Learning and Memory of Homozygous Cln3^Δex7/8^ Mice

**Figure 3:**
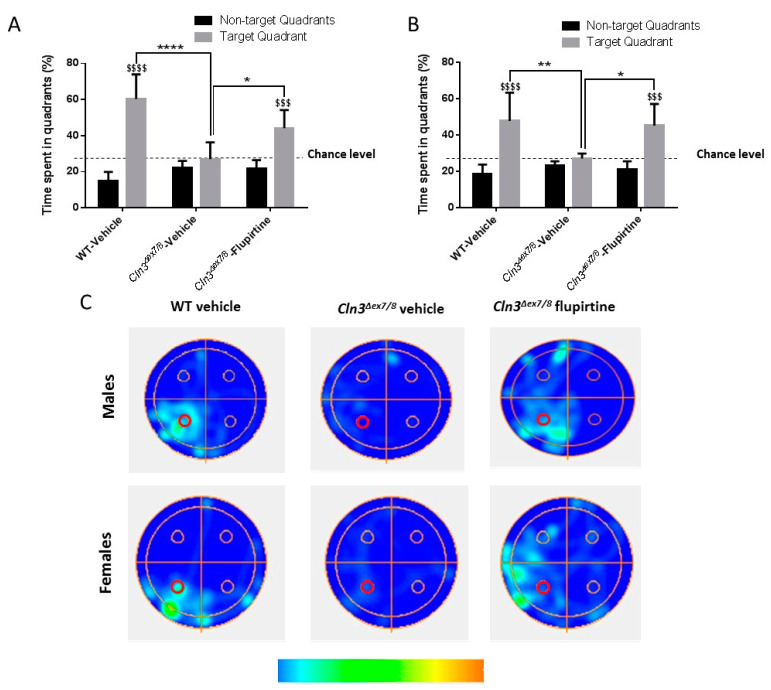
Impact of flupirtine on learning and memory of male and female *Cln3^Δex7/8^* mice. Percentage of time spent in target vs. non-target quadrant of (**A**) male and (**B**) female mice in the different groups (vehicle-treated WT, vehicle-treated *Cln3^Δex7/8^*, and flupirtine-treated *Cln3^Δex7/8^*) during the probe test (*n* = 8 per group). All data are expressed as mean ± SEM. ^$^ *p*: Target quadrant compared to non-target quadrant; and * *p*: compared to vehicle-treated WT mice. * *p* < 0.05, ** *p* < 0.01, **** *p* < 0.0001, ^$$$^ *p* < 0.001, and ^$$$$^ *p* < 0.0001. (**C**) Representative heat maps of swimming paths of one mouse from different groups during the probe test in male and female mice. An empty, bolded red circle indicates location of the target platform (ᴏ).

The Morris water maze (MWM) is a test used to assess cognitive function, more specifically, spatial learning and memory. The ‘probe trial’, in which the platform was removed, was used to assess spatial memory for the previously learned platform location. The time spent in the target quadrant compared to the average time spent in the non-target quadrants is an indicator of the animal’s recall of platform location. WT male and female mice spent significantly more time in the target quadrant as compared to the average time spent in the non-target quadrants, suggestive of recall of platform location (Figure 3A–C). Vehicle-treated *Cln3^Δex7/8^* male and female mice spent significantly less time than their WT male littermates in the target quadrant (Figure 3A–C). Flupirtine treatment improved memory retention in *Cln3^Δex7/8^* male and female mice, with a significant increase in time spent in the target quadrant compared to vehicle-treated *Cln3^Δex7/8^* mice, suggestive of recall of platform location (Figure 3A–C).

### 3.3. Impact of Flupirtine on Anti-Apoptotic Gene Expression in Male and Female Cln3^Δex7/8^ Mice

**Figure 4:**
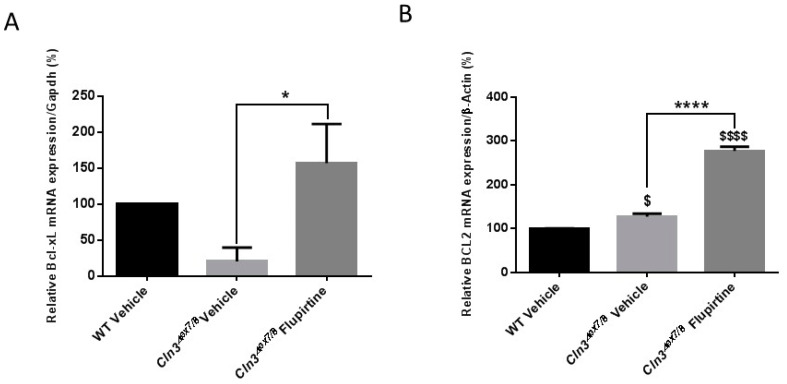
Mouse brain gene expression of anti-apoptotic markers. (**A**) *Bcl-xl* gene expression levels in the brain of vehicle-treated WT, vehicle-treated *Cln3^Δex7/8^*, and flupirtine-treated *Cln3^Δex7/8^* male mice (*n* = 4 per group). Data are expressed as mean ± SEM, * *p*: compared to *Cln3^Δex7/8^* vehicle-treated mice. * *p* < 0.05; (**B**) Bcl-2 gene expression levels in the brain of vehicle-treated WT, vehicle-treated *Cln3^Δex7/8^*, and flupirtine-treated *Cln3^Δex7/8^* female mice (*n* = 4 per group). Data are expressed as mean ± SEM. ^$^ *p*: compared to vehicle-treated WT mice, and, * *p*: compared to vehicle-treated *Cln3^Δex7/8^* mice. **** *p* < 0.0001, ^$^ *p* < 0.05, and ^$$$$^ *p* < 0.0001.

The expression of several anti-apoptotic (*Bcl-2, Bcl-xL, Akt, Xiap*) and pro-apoptotic genes (*Fadd, Cytochrome C, Caspase 3, Caspase 6, Caspase 9, Apaf-1, Bad, Bax*) were measured in male and female mice. The anti-apoptotic gene B-cell lymphoma extra-large (*Bcl-xl*) gene expression level was downregulated in vehicle-treated *Cln3^Δex7/8^* mice compared to vehicle-treated WT male mice (Figure 4A). Flupirtine treatment had a significant effect only on *Bcl-xl* expression levels, by increasing mRNA expression level in *Cln3^Δex7/8^* male mice (Figure 4A). In female mice, B-cell lymphoma 2 (*Bcl-2*) gene expression was slightly higher in vehicle-treated *Cln3^Δex7/8^* mice compared to vehicle-treated WT female mice (Figure 4B). Flupirtine, however, significantly increased only the mRNA expression level of *Bcl-2* in *Cln3^Δex7/8^* compared to vehicle-treated *Cln3^Δex7/8^* mice (Figure 4B). The expression level of other anti-apototic and pro-apototic genes remained unchanged (data not shown).

### 3.4. Impact of Flupirtine on Gene Expression of Enzymes of Ceramide Metabolism in Cln3^Δex7/8^ Mice

**Figure 5:**
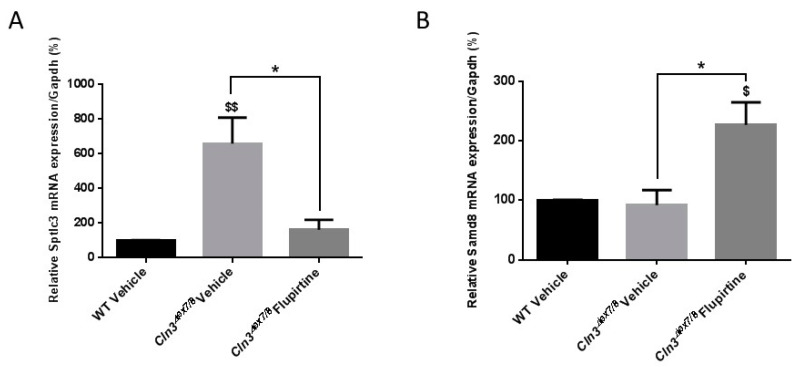
Mouse brain gene expression of ceramide synthesis/degradation enzymes. (**A**) Sptlc3 gene expression levels in the brain of vehicle-treated WT, vehicle-treated *Cln3^Δex7/8^*, and flupirtine-treated *Cln3^Δex7/8^* male mice (*n* = 4 per group). Data are expressed as mean ± SEM. ^$^ *p*: compared to WT vehicle-treated mice, and, * *p*: compared to vehicle-treated *Cln3^Δex7/8^* mice. * *p* < 0.05, and ^$$^ *p* < 0.01; (**B**) Samd8 gene expression levels in the brain of vehicle-treated WT, vehicle-treated *Cln3^Δex7/8^*, and flupirtine-treated *Cln3^Δex7/8^* female mice (*n* = 4 per group). Data are expressed as mean ± SEM. ^$^ *p*: compared to vehicle-treated WT mice, and, * *p*: compared to vehicle-treated *Cln3^Δex7/8^* mice. * *p* < 0.05 and ^$^ *p* < 0.05.

Several ceramide synthesis enzymes (*Sptlc2, Sptlc3, Kdsr, CerS1-6, Degs1, Degs2, Smpd2, Smpd3, Gba, GalC*) and ceramide degradation enzymes (*Asah1, Asah2, Samd8, Ugcg, Ugt8*) were investigated in male and female mice. Serine palmitoyltransferase 3 (*Sptlc3*) levels were significantly upregulated in vehicle-treated *Cln3^Δex7/8^* mice compared to vehicle-treated WT male mice (Figure 5A). Flupirtine treatment had a significant impact only on the expression level of key ceramide synthesis enzyme, Sptlc3, in the de novo pathway, by reducing mRNA expression levels compared to *Cln3^Δex7/8^* vehicle-treated male mice (Figure 5A). In female mice, sterile alpha motif domain containing 8 (*Samd8*) gene expression level did not differ between vehicle-treated WT and vehicle-treated *Cln3^Δex7/8^* female mice. *Samd8* is an endoplasmic reticulum (ER) transferase that converts phosphatidylethanolamine (PE) and ceramide to ceramide phosphoethanolamine (CPE). Flupirtine significantly increased expression levels of only *Samd8* compared to vehicle-treated WT and to *Cln3^Δex7/8^* female mice (Figure 5B). The expression level of other ceramide synthesis/degradation enzymes remained unchanged (data not shown).

### 3.5. Effect of Flupirtine Supplementation on Astrocytosis in Cln3^Δex7/8^ Mouse Brains

**Figure 6:**
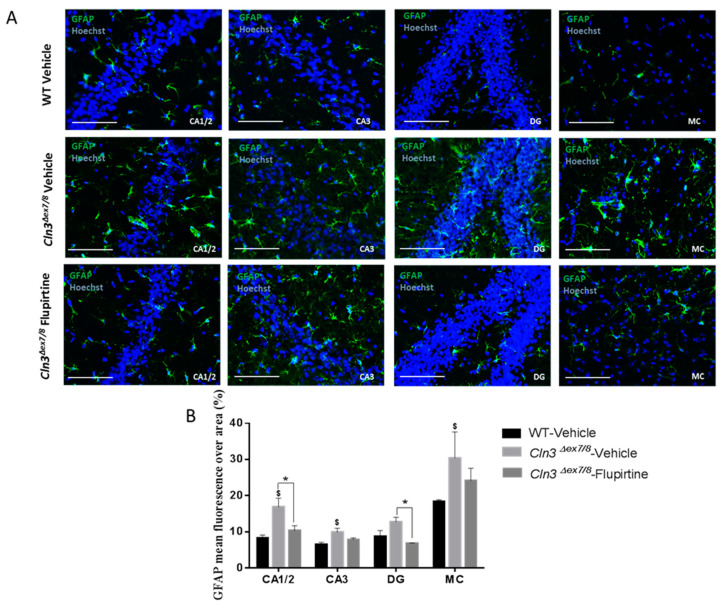
Impact of flupirtine supplementation on astrocytosis in hippocampus and motor cortex of male *Cln3^Δex7/8^* mice. (**A**) Representative images of hippocampus regions CA1, CA2, CA3 and dentate gyrus (DG), as well as primary motor cortex from vehicle-treated WT, vehicle-treated *Cln3^Δex7/8^*, and flupirtine-treated *Cln3^Δex7/8^* male mice stained with GFAP in green and counterstained with Hoechst in blue (*n* = 4; Scale bars = 20 μm).; (**B**) Glial fibrillary acidic protein (GFAP) mean fluorescence over area in vehicle-treated WT, vehicle-treated *Cln3^Δex7/8^*, and flupirtine-treated *Cln3^Δex7/8^* male mice in hippocampus regions CA1, CA2, CA3 and dentate gyrus (DG), as well as in primary motor cortex (MC). Data are expressed as mean ± SEM. ^$^ *p* < 0.05 compared to vehicle-treated WT, * *p* < 0.05 compared to vehicle-treated *Cln3^Δex7/8^*.

Fluorescence microscopy demonstrated significant enhanced GFAP immunostaining (green) in vehicle-treated *Cln3^Δex7/8^* male mice relative to WT littermates in CA1/2 and CA3 hippocampus, dentate gyrus (DG), and motor cortex (MC) (Figure 6A,B). Treatment with flupirtine decreased glial activation in all brain regions studied in *Cln3^Δex7/8^* male mice (Figure 6A,B). Results were statistically significant only in CA1/2 hippocampus, and motor cortex (MC). Hoechst staining (blue) indicates diminished number of hippocampal neurons in vehicle-treated *Cln3^Δex7/8^* versus WT, and also an increase in neurons in flupirtine-treated male mice versus vehicle-treated *Cln3^Δex7/8^* male mice (Figure 6A).

**Figure 7:**
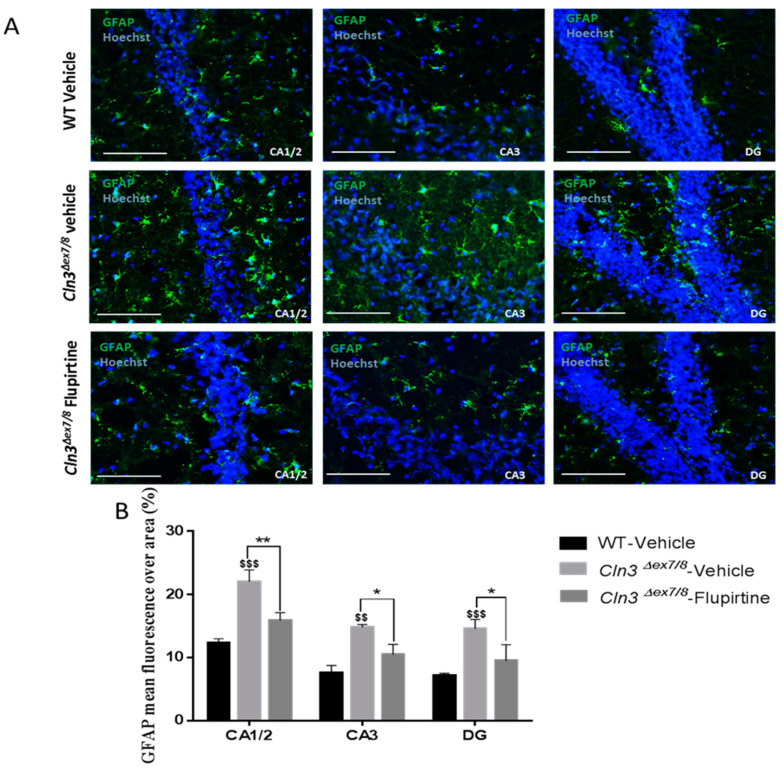
Impact of flupirtine supplementation on astrocytosis in hippocampus and motor cortex of female *Cln3^Δex7/8^* mice. (**A**) Representative images of hippocampus regions CA1, CA2, CA3 and dentate gyrus (DG), from vehicle-treated WT, vehicle-treated *Cln3^Δex7/8^*, and flupirtine-treated *Cln3^Δex7/8^* female mice stained with GFAP in green and counterstained with Hoechst in blue (*n* = 4; scale bars = 20 μm).; (**B**) glial fibrillary acidic protein (GFAP) mean fluorescence over area in vehicle-treated WT, vehicle-treated *Cln3^Δex7/8^*, and flupirtine-treated *Cln3^Δex7/8^* male mice in hippocampus regions CA1, CA2, CA3 and dentate gyrus (DG), as well as in primary motor cortex (MC). Data are expressed as mean ± SEM. ^$^ *p:* compared to vehicle-treated WT, * *p*: compared to vehicle-treated *Cln3^Δex7/8^*. * *p* < 0.05, ** *p* < 0.01, ^$$^ *p* < 0.01, and ^$$$^ *p* < 0.001.

In females, GFAP immunostaining was significantly enhanced in vehicle-treated *Cln3^Δex7/8^* mice relative to their WT female littermates in CA1/2 and CA3 hippocampus, as well as in DG (Figure 7A,B). Treatment with flupirtine significantly decreased astrogliosis in CA1/2 and CA3 hippocampus, and DG in *Cln3^Δex7/8^* female mice (Figure 7A,B). Also, note an increase in the number of blue, Hoechst-stained neurons in hippocampus and dentate gyrus in both wild type and flupirtine-treated *Cln3^Δex7/8^* female mice compared to vehicle-treated *Cln3^Δex7/8^* female mice (Figure 7A). In motor cortex (MC) of females, no significant difference in GFAP levels was observed (data not shown).

### 3.6. Impact of Flupirtine Supplementation on Neuronal Cell Counts in Cln3^Δex7/8^ Brains

**Figure 8:**
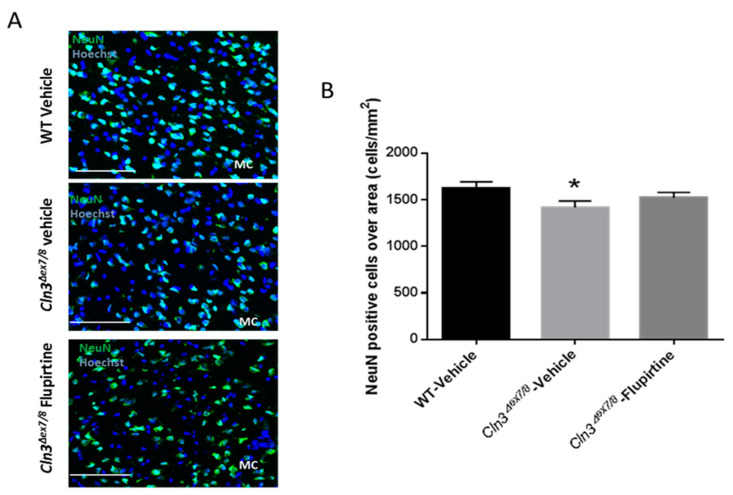
Effect of flupirtine treatment on motor cortex neuron numbers in *Cln3^Δex7/8^* mice. (**A**) Representative images of primary motor cortex from vehicle-treated WT, vehicle-treated *Cln3^Δex7/8^* and flupirtine-treated *Cln3^Δex7/8^* male mice stained with NeuN (mature neuronal marker) in green and counterstained with Hoechst in blue (*n* = 4; Scale bars = 50 μm).; (**B**) NeuN-positive cells normalized to area in the primary motor cortex (MC) of vehicle-treated WT, vehicle-treated *Cln3^Δex7/8^*, and flupirtine-treated *Cln3^Δex7/8^* male mice. Data are expressed as mean ± SEM. * *p* < 0.05 compared to vehicle-treated WT.

The number of NeuN-positive cells decreased significantly in the MC of vehicle-treated *Cln3^Δex7/8^* male mice versus vehicle-treated WT mice (Figure 8A,B). Although not significant, NeuN-stained cells in MC of flupirtine-treated *Cln3^Δex7/8^* male mice compared to vehicle-treated *Cln3^Δex7/8^* mice showed a slight increase, close to the level in WT mice (Figure 8A,B). No significant difference in the neuronal cell counts of female MC was seen (data not shown).

### 3.7. Impact of Compound 6 Supplementation on Behavioral Parameters

**Figure 9:**
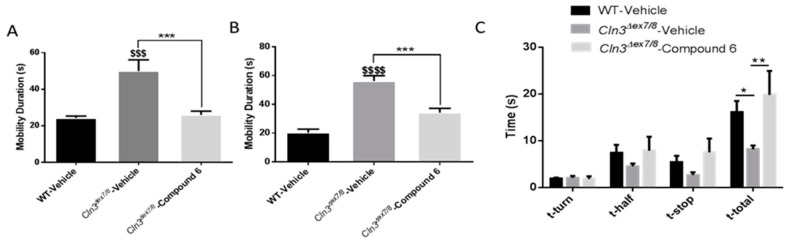
Impact of compound 6 treatment in *Cln3^Δex7/8^* mice. Open field behavioral parameter (mobility duration) in vehicle-treated WT, vehicle-treated *Cln3^Δex7/8^*, and compound 6-treated *Cln3^Δex7/8^* (**A**) male and (**B**) female mice (*n* = 8 per group). Data are expressed as mean ± SEM. ^$^ *p*: compared to WT vehicle-treated mice, and, * *p*: compared to vehicle-treated *Cln3^Δex7/8^* mice. *** *p* < 0.001, ^$$$^ *p* < 0.001, and ^$$$$^ *p* < 0.0001. (**C**) Pole climbing test in vehicle-treated WT, vehicle-treated *Cln3^Δex7/8^*, and flupirtine-treated *Cln3^Δex7/8^* male mice (*n* = 8 per group). Time to turn (t_turn_), time needed to reach center of pole (t_1/2_), amount of time stopped (t_stop_), and total time to descend (t_total_) were recorded. Data are expressed as mean ± SEM. * *p* < 0.05, ** *p* < 0.01.

In the open field test, compound 6 significantly slowed down locomotor hyperactivity of male and female *Cln3^Δex7/8^* mice (Figure 9A,B). Similarly, a similar effect was seen in the pole-climbing test, where compound 6 significantly increased time needed by male mice to descend the pole compared to vehicle treated mice (Figure 9C).

## 4. Discussion

This study addresses novel small molecule treatment strategies for CLN3 disease in a *Cln3^Δex7/8^* knock-in mouse model. Hopefully, this will translate into future knowledge to improve the lives of CLN3 patients.

Flupirtine is well known for its significant powerful anti-oxidative, anti-apoptotic, and neuroprotective effects in vitro and in vivo. The effectiveness of the chosen daily dose of flupirtine (30 mg/kg per os) has been demonstrated in testing for anti-nociceptive, anticonvulsant, and anti-apoptotic activity in rodents [23,24,25,26,27]. This study is the first to evaluate the use of flupirtine as potential treatment for CLN3 disease in an animal model, i.e., in vivo. Previous studies proved that flupirtine reaches brain regions, including hippocampus and cortex, and that it rapidly crosses the blood brain barrier and enters other tissues. The liver is the primary organ responsible for its metabolism [15].

Behavioral tests assessed different aspects of *Cln3^Δex7/8^* mouse motor strength, coordination, balance, as well as learning and spatial memory functions before and after flupirtine treatment. Male and female vehicle-treated *Cln3^Δex7/8^* mice exhibit significantly increased mobility with respect to WT controls in the open field test. The hyperactive phenotype prominent in vehicle-treated *Cln3^Δex7/8^* mice is described for the first time here in mice at of 18 weeks of age. This was not previously documented in *Cln3^Δex7/8^* mice at 40 weeks of age [28]. This suggests that, at a young age, *Cln3^Δex7/8^* mice express a distinct behavioral phenotype prior to onset of more severe CLN3 disease symptoms, including motor decline. *Cln3^Δex7/8^* mice also showed increased random and chaotic exploratory activity compared to WT controls in a novel environment. This indicates inattentiveness and diminished executive function. Treatment with flupirtine significantly attenuated this abnormal mobility in male and female *Cln3^Δex7/8^* mice. This phenotype may be consistent with the notion that diminished executive function of vehicle-treated *Cln3^Δex7/8^* mice is driving their inattentive hyperactivity. The vertical pole test evaluates spatial and motor orientation and balance of mice [29]. Reduced latency of vehicle-treated *Cln3^Δex7/8^* mice to descend the pole is explained by their quick and less controlled behavior compared to WT animals, consistent with open field behavioral assessments of excessive mobility. The aforementioned behavioral test reveals prominent hyperactivity and attentional deficits in vehicle-treated *Cln3^Δex7/8^* mice, while treatment with flupirtine lessened this impulsive phenotype. These results are in line with other mouse models of neurodevelopmental psychiatric disorder of attention-deficit hyperactivity disorder (ADHD). Mice recapitulating this disease are characterized by impulsivity, inattentiveness, and hyperactivity [30]. Stress has a direct profound effect on rodent behavior and physical activity [31]. Chronic stress leads to increased corticosterone levels [31], as observed in WT male and female mice with respect to *Cln3^Δex7/8^* mice. Several studies elucidate that animals who do not experience stress show higher exploration, locomotion, and physical activity in an open field test, as a reaction to an unknown environment [32,33]. Therefore, the markedly decreased corticosterone levels in serum of *Cln3^Δex7/8^* mice with respect to WT animals explains their hyperactivity resulting from reduced stress levels in male and female *Cln3^Δex7/8^* mice. Learning and cognitive ability of mice was tested at 16 weeks of age using the Morris water maze (MWM) test. Analysis of the swim paths of mice during the probe trial showed that flupirtine-treated male and female *Cln3^Δex7/8^* mice adopted strategies and maintained spatial preference in the target quadrant, contrary to vehicle-treated *Cln3^Δex7/8^* mice that show coverage of the whole maze. Flupirtine significantly enhanced spatial learning, navigation and memory retention in *Cln3^Δex7/8^* male and female mice.

Apoptosis is a naturally-occurring mechanism of cell death and helps maintain tissue homeostasis [34]. Neuronal cell loss is evident in brain sections from post-mortem CLN3 disease patients [3]. Numerous apoptotic cells are present within cortical brain sections from CLN3 disease patients [3]. CLN3 patient-derived lymphoblasts have decreased growth rate compared to normal lymphoblasts, validating that apoptosis is one of the mechanisms implicated in CLN3 disease pathogenesis [35]. Other studies demonstrate damage and apoptosis of neuronal and glial cells in hippocampus and cortex of CLN3 patients in addition to marked loss of cortical neurons due to apoptotic cell death [6]. CLN3 defects also perturb calcium signaling, leading to a profound defects in neuronal survival [36].

Most neuronal death in CLN2 and CLN3 brains takes place via apoptosis, and the surviving neurons upregulate Bcl-2 [5]. Treatment with flupirtine significantly upregulated expression of anti-apoptotic BCL-2 in CLN3-deficient cells in vitro [20]. This is the case *in vivo* in this current study as flupirtine-treated *Cln3^Δex7/8^* female mice show a remarkable increase in *Bcl-2* expression. In males, another anti-apoptotic protein, *Bcl-xl*, was upregulated following treatment with flupirtine. Different proteins were impacted in male versus female mice, yet the end result was upregulation of anti-apoptotic pathways and hence, reduction of cell death in brains of *Cln3^Δex7/8^* mice given flupirtine. This variation among sexes is not a new observation in this disease. We documented this in another study using exogenous galactosylceramide as potential treatment for CLN3 disease [28].

CLN3 is directly implicated in apoptotic cell death signaling cascades by activating caspase-dependent and caspase-independent pathways [8]. Ceramide is a major sphingolipid second messenger implicated in several cell processes and impacts divergent pathways [37]. Sphingolipids are major bioactive lipids involved in homeostasis, growth, proliferation and cell death [38]. Ceramide mediates anti-proliferative events, such as apoptosis, growth inhibition, cell differentiation, and senescence [10]. This biomolecule possesses complex biophysical properties and acts as a central hub. Regulation of its levels affects catabolism and break-down of various sphingolipid species [10]. Ceramide is generated through several complex interrelated pathways either via the de novo pathway, sphingomyelin, or cerebroside catabolism. Ceramide is synthesized de novo in the endoplasmic reticulum or through breakdown of sphingomyelin in Golgi, plasma membrane, or mitochondrial membrane [39]. Defects in ceramide signaling pathways often result in augmenting programmed cell death in multiple cell types, including neurons [40]. Previous studies show that ceramide levels are increased in CLN3-deficient cells and brain of CLN3 patients [11]. Published reports demonstrate that 17 week-old *Cln3^Δex7/8^* mice express higher levels of ceramide in brain compared to age-matched WT mice [14]. Sptlc3 catalyzes the initial steps in formation of ceramide via the de novo pathway by condensing serine and palmitoyl Co-A to generate 3-ketoshphinganine (3-KDS) [10]. We documented decreased *Sptlc3* levels in flupirtine-treated *Cln3^Δex7/8^* mice versus vehicle-treated *Cln3^Δex7/8^* male mice. Downregulation of *Sptlc3* leads to diminution of ceramide generation via the de novo ceramide pathway. As for the other enzymes of the de novo pathway, the expression of *Sptlc2* and *Degs1* also decreased with flupirtine treatment, but did not reach significance (data not shown). This supports our conclusion that flupirtine treatment in male mice impacts the de novo synthesis pathway. In females, however, a different pathway in ceramide signaling is at play. *Samd8* is an ER transferase that converts phosphatidylethanolamine (PE) and ceramide to ceramide phosphoethanolamine (CPE). *Samd8* levels are increased in flupirtine-treated *Cln3^Δex7/8^* female mice. *Samd8* operates as a ceramide sensor to control ceramide homeostasis in the ER rather than a converter of ceramides. This implicates that the ceramide salvage pathway is modulated in flupirtine-treated *Cln3^Δex7/8^* female mice with the end result of diminished ceramide levels. Empirical evidence from our study confirms reduced synthesis (decreased *Sptlc3* expression) in flupirtine-treated male mice, but increased degradation of ceramide (increased *Samd8* expression) in female flupirtine-treated mice. Physiologic gender differences affects drug activity and charactersitics, including pharmacokinetics [41]. Differences in body size result in larger distribution volumes, faster total clearance, and less tissue absorption of some medications in men compared to women [42]. This may explain higher brain absorption in female compared to male mice. Moreover, sex hormones, in females, have a direct effect on drug absorption, distribution, metabolism, elimination and adverse effects [43].This study implies that sex-specific drug dosing regimens may be warranted for treatment of neurological diseases that affect the blood brain barrier, including CLN3 disease in mouse and man.

Activation of the glial cell population contributes to imbalance in CNS function and impacts cognitive function [44]. Hyperactive astrocytes are observed in neurodegenerative disorders and following brain injury documented by GFAP as biomarker. In males, microscopic inspection of vehicle-treated *Cln3^Δex7/8^* mouse brain documents widespread intracellular GFAP staining in hippocampal regions (CA1, CA2, CA3, and the dentate gyrus) and in the MC relative to age-matched, vehicle-treated WT animals. Mice treated with flupirtine had significantly lower levels of GFAP staining in these regions. This data provides evidence that flupirtine attenuates astrogliosis at the level of the hippocampus and MC in *Cln3^Δex7/8^* male mice. In females, flupirtine was able to attenuate GFAP immunostaining in CA1/2, CA3 and DG regions of the hippocampus. The motor cortex did not show any difference in vehicle-treated *Cln3^Δex7/8^* female mice compared to WT. This may explain better performance in *Cln3^Δex7/8^* female mice on the rotarod compared to males as it tests motor skills (data not shown).

The neuronal nuclear protein (NeuN) is a marker not detected in glial cells or other cells in the brain [28]. NeuN assesses neuronal health and loss of this protein is indicative of damage. Neuronal cell loss in CLN3 patients is at the root of CLN3 disease pathogenesis [45]. NeuN-positive cell were significantly ablated in vehicle-treated *Cln3^Δex7/8^* mouse. Although it did not reach significance, flupirtine resulted in an increase in the neuronal population in motor cortex (MC) of male mice. This suggests that flupirtine conferred neuroprotection and reduced cell death in brains of *Cln3^Δex7/8^* mice. In females, there was no difference in neuronal counts in motor cortex of vehicle-treated *Cln3^Δex7/8^* female mice compared to WT. In a previous study, affected female mice 44 weeks of age did show a diminution in NeuN positive cells [28], suggesting that unlike males, neuronal loss in females starts at a later age.

The novel, flupirtine-like allyl carbamate derivative, compound 6, developed to possess physicochemical properties desirable for CNS therapeutics had an improved Multiparameter optimization (MPO) score. The latter predicts blood–brain barrier (BBB) penetration, an essential parameter for neuroprotective compounds, and was ≥ 4 more effective [19]. The newly synthesized allyl carbamate derivative of flupirtine, compound 6, showed potential for improved neuroprotection, after screening in vitro nine flupirtine derivatives [20]. Here, we report early promising behavioral in vivo results for compound 6 for treatment of *Cln3^Δex7/8^* mice. Although pharmacokinetics and toxicological safety remain to be established for compound 6, the promising behavioral data obtained in this study are worth reporting. Treatment with compound 6 significantly attenuated the high mobility documented by open field and pole climbing in *Cln3^Δex7/8^* mice, suggesting more work is necessary to determine optimal dosing for this compound.

## 5. Conclusions

In conclusion, these findings suggest that flupirtine, and compound 6, improve neurobehavioral measures. Flupirtine impacted ceramide biosynthesis and apoptotic signaling pathways. Flupirtine affected a broad-spectrum of actionable targets, providing insights into the pathobiology of CLN3 disease in humans, particularly uncovering impact on gender-specific signaling pathways. Flupirtine shows promise in males and females, and the allyl carbamate derivative, compound 6, needs further preclinical analyses and development. These findings extend our knowledge of the role of drugs in the treatment of a fatal pediatric neurodegenerative disease implying more work lies ahead for development into clinically applicable therapies for CLN3 patients.

## 6. Patents

R.-M.B. has an Application for Method of Treating Batten Disease. Inventor: Rose-Mary Boustany. Duke (File No. 5405-240 PR). US Patent and Trademark No. 10/148,859 (U.S. National Phase); Use Patent issued 11/23/2004 US Patent # 6 821 995, expired 11/23/2014.

N.K., P.C.T. and R.-M.B. are inventors on a patent application detailing the aromatic carbamates described herein: ‘Functionalized Pyridine Carbamates with enhanced Neuroprotective Activity’ PCT Int. Appl. (2019), WO2019014547 A8.

## Figures and Tables

**Table 1:** Mouse cDNA primer sequences.

Gene Name	Primer Sequence (F = forward, R = reverse)
*Bcl-xL F*	AGGTTCCTAAGCTTCGCAATTC
*Bcl-xL R*	TGTTTAGCGATTCTCTTCCAGG
*BCL-2 F*	TGTGTGTGGAGAGCGTCAAC
*BCL-2 R*	TGAGCAGAGTCTTCAGAGAC
*Sptlc3 F*	TGATTTCTCTCCGGTGATCC
*Sptlc3 R*	GGAAATCCAACAACCACCAC
*Samd8 F*	ATCACATTGCTCACGCTGAC
*Samd8 R*	GCAATTTTCGGACTGAGAGC
*β-actin F*	ACACTGTGCCCATCTACGAG
*β-actin R*	ATTTCCCTCTCAGCTGTGGT
*Gapdh F*	TGTTCCTACCCCCAATGTGT
*Gapdh R*	AGTTGCTGTTGAAGTCGCAG

## References

[1] Jalanko A., Braulke T. (2009). Neuronal ceroid lipofuscinoses. Biochim. Biophys. Acta (BBA) Mol. Cell Res..

[2] Persaud-Sawin D.-A., Mousallem T., Wang C., Zucker A., Kominami E., Boustany R.-M.N. (2007). Neuronal Ceroid Lipofuscinosis: A Common Pathway?. Pediat. Res..

[3] Lane S.C., Jolly R.D., Schmechel D.E., Alroy J., Boustany R.-M. (1996). Apoptosis as the Mechanism of Neurodegeneration in Batten’s Disease. J. Neurochem..

[4] Munroe P.B., Mitchison H.M., O’Rawe A.M., Anderson J.W., Boustany R.M., Lerner T.J., Taschner P.E., de Vos N., Breuning M.H., Gardiner R.M. (1997). Spectrum of mutations in the Batten disease gene, CLN3. Am. J. Hum. Genet..

[5] Puranam K., Qian W.H., Nikbakht K., Venable M., Obeid L., Hannun Y., Boustany R.M. (1997). Upregulation of Bcl-2 and elevation of ceramide in Batten disease. Neuropediatrics.

[6] Pontikis C.C., Cotman S.L., MacDonald M.E., Cooper J.D. (2005). Thalamocortical neuron loss and localized astrocytosis in the Cln3Δex7/8 knock-in mouse model of Batten disease. Neurobiol. Dis..

[7] Lerner T.J., Boustany R.-M.N., Anderson J.W., D’Arigo K.L., Schlumpf K., Buckler A.J., Gusella J.F., Haines J.L. (1995). Isolation of a novel gene underlying batten disease, *CLN3*. Cell.

[8] Persaud-Sawin D.A., Boustany R.M.N. (2005). Cell death pathways in juvenile Batten disease. Apoptosis.

[9] Guo W.-X., Mao C., Obeid L.M., Boustany R.-M. (1999). A Disrupted Homologue of the Human CLN3 or Juvenile Neuronal Ceroid Lipofuscinosis Gene in Saccharomyces cerevisiae: A Model to Study Batten Disease. Cell. Mol. Neurobiol..

[10] Ogretmen B., Hannun Y.A. (2004). Biologically active sphingolipids in cancer pathogenesis and treatment. Nat. Rev. Cancer.

[11] Puranam K.L., Guo W.-X., Qian W.-H., Nikbakht K., Boustany R.-M. (1999). CLN3 Defines a Novel Antiapoptotic Pathway Operative in Neurodegeneration and Mediated by Ceramide. Mol. Genet. Metab..

[12] Rusyn E., Mousallem T., Persaud-Sawin D.-A., Miller S., Boustany R.-M.N. (2008). CLN3p Impacts Galactosylceramide Transport, Raft Morphology, and Lipid Content. Pediat. Res..

[13] Dhar S., Bitting R.L., Rylova S.N., Jansen P.J., Lockhart E., Koeberl D.D., Amalfitano A., Boustany R.-M.N. (2002). Flupirtine blocks apoptosis in batten patient lymphoblasts and in human postmitotic CLN3- and CLN2-deficient neurons. Ann. Neurol..

[14] El-Sitt S., Soueid J., Al Ali J., Makoukji J., Makhoul N.J., Harati H., Boustany R.-M. (2019). Developmental Comparison of Ceramide in Wild-Type and Cln3 (Δex7/8) Mouse Brains and Sera. Front. Neurol..

[15] Patil A.M., Matter A.B., Raol H.Y., Bourne W.A.D., Kelley A.R., Kompella B.U. (2018). Brain Distribution and Metabolism of Flupirtine, a Nonopioid Analgesic Drug with Antiseizure Effects, in Neonatal Rats. Pharmaceutics.

[16] Perovic S., Pergande G., Ushijima H., Kelve M., Forrest J., Müller W.E.G. (1995). Flupirtine Partially Prevents Neuronal Injury Induced by Prion Protein Fragment and Lead Acetate. Neurodegeneration.

[17] Müller W.E.G., Laplanche J.-L., Ushijima H., Schröder H.C. (2000). Novel approaches in diagnosis and therapy of Creutzfeldt–Jakob disease. Mech. Ageing Dev..

[18] Devulder J. (2010). Flupirtine in Pain Management. CNS Drugs.

[19] Kinarivala N., Patel R., Boustany R.-M., Al-Ahmad A., Trippier P.C. (2017). Discovery of Aromatic Carbamates that Confer Neuroprotective Activity by Enhancing Autophagy and Inducing the Anti-Apoptotic Protein B-Cell Lymphoma 2 (Bcl-2). J. Med. Chem..

[20] Makoukji J., Saadeh F., Mansour K.A., El-Sitt S., Al Ali J., Kinarivala N., Trippier P.C., Boustany R.-M. (2018). Flupirtine derivatives as potential treatment for the neuronal ceroid lipofuscinoses. Ann. Clin. Transl. Neurol..

[21] Kinarivala N., Trippier P.C. (2016). Progress in the Development of Small Molecule Therapeutics for the Treatment of Neuronal Ceroid Lipofuscinoses (NCLs). J. Med. Chem..

[22] Seibenhener M.L., Wooten M.C. (2015). Use of the Open Field Maze to measure locomotor and anxiety-like behavior in mice. J. Vis. Exp..

[23] Huang P., Li C., Fu T., Zhao D., Yi Z., Lu Q., Guo L., Xu X. (2015). Flupirtine attenuates chronic restraint stress-induced cognitive deficits and hippocampal apoptosis in male mice. Behav. Brain Res..

[24] Jaeger H.M., Pehlke J.R., Kaltwasser B., Kilic E., Bähr M., Hermann D.M., Doeppner T.R. (2015). The indirect NMDAR inhibitor flupirtine induces sustained post-ischemic recovery, neuroprotection and angioneurogenesis. Oncotarget.

[25] Kumar M., Gokul C.G., Somashekar H.S., Adake P., Acharya A., Santhosh R. (2011). Anticonvulsant activity of flupirtine in Albino mice. Pharmacologyonline.

[26] Morecroft I., Murray A., Nilsen M., Gurney A.M., MacLean M.R. (2009). Treatment with the Kv7 potassium channel activator flupirtine is beneficial in two independent mouse models of pulmonary hypertension. Br. J. Pharm..

[27] Nickel B. (1987). The antinociceptive activity of flupirtine: A structurally new analgesic. Postgrad. Med. J..

[28] El-Sitt S., Soueid J., Maalouf K., Makhoul N., Al Ali J., Makoukji J., Asser B., Daou D., Harati H., Boustany R.-M. (2019). Exogenous Galactosylceramide as Potential Treatment for CLN3 Disease. Ann. Neurol..

[29] Justice J.N., Carter C.S., Beck H.J., Gioscia-Ryan R.A., McQueen M., Enoka R.M., Seals D.R. (2014). Battery of behavioral tests in mice that models age-associated changes in human motor function. Age.

[30] Bouchatta O., Manouze H., Bouali-benazzouz R., Kerekes N., Ba-M’hamed S., Fossat P., Landry M., Bennis M. (2018). Neonatal 6-OHDA lesion model in mouse induces Attention-Deficit/Hyperactivity Disorder (ADHD)-like behaviour. Sci. Rep..

[31] DeVallance E., Riggs D., Jackson B., Parkulo T., Zaslau S., Chantler P.D., Olfert I.M., Bryner R.W. (2017). Effect of chronic stress on running wheel activity in mice. PLoS ONE.

[32] Bondar N.P., Lepeshko A.A., Reshetnikov V.V. (2018). Effects of Early-Life Stress on Social and Anxiety-Like Behaviors in Adult Mice: Sex-Specific Effects. Behav. Neurol..

[33] Borkar C.D., Dorofeikova M., Le Q.E., Vutukuri R., Vo C., Hereford D., Resendez A., Basavanhalli S., Sifnugel N., Fadok J.P. (2020). Sex differences in behavioral responses during a conditioned flight paradigm. Behav. Brain Res..

[34] Gelbard H.A., Boustany R.M., Schor N.F. (1997). Apoptosis in development and disease of the nervous system: II. Apoptosis in childhood neurologic disease. Pediat. Neurol..

[35] Persaud-Sawin D.A., VanDongen A., Boustany R.M. (2002). Motifs within the CLN3 protein: Modulation of cell growth rates and apoptosis. Hum. Mol. Genet..

[36] Bosch M.E., Kielian T. (2019). Astrocytes in juvenile neuronal ceroid lipofuscinosis (CLN3) display metabolic and calcium signaling abnormalities. J. Neurochem..

[37] Jayadev S., Liu B., Bielawska A.E., Lee J.Y., Nazaire F., Pushkareva M.Y., Obeid L.M., Hannun Y.A. (1995). Role for Ceramide in Cell Cycle Arrest. J. Biol. Chem..

[38] Bilal F., Montfort A., Gilhodes J., Garcia V., Riond J., Carpentier S., Filleron T., Colacios C., Levade T., Daher A. (2019). Sphingomyelin Synthase 1 (SMS1) Downregulation Is Associated With Sphingolipid Reprogramming and a Worse Prognosis in Melanoma. Front. Pharmacol..

[39] Persaud-Sawin D.A., McNamara J.O., Rylova S., Vandongen A., Boustany R.M. (2004). A galactosylceramide binding domain is involved in trafficking of CLN3 from Golgi to rafts via recycling endosomes. Pediat. Res..

[40] Di Pardo A., Maglione V. (2018). Sphingolipid Metabolism: A New Therapeutic Opportunity for Brain Degenerative Disorders. Front. Neurosci..

[41] Whitley H., Lindsey W. (2009). Sex-based differences in drug activity. Am Fam Physician.

[42] Schwartz J.B. (2003). The influence of sex on pharmacokinetics. Clin. Pharm..

[43] Spoletini I., Vitale C., Malorni W., Rosano G.M. (2012). Sex differences in drug effects: Interaction with sex hormones in adult life. Handb. Exp. Pharm..

[44] Becerra-Calixto A., Cardona-Gómez G.P. (2017). The Role of Astrocytes in Neuroprotection after Brain Stroke: Potential in Cell Therapy. Front. Mol. Neurosci..

[45] Braak H., Goebel H.H. (1978). Loss of pigment-laden stellate cells: A severe alteration of the isocortex in juvenile neuronal ceroid-lipofuscinosis. Acta Neuropathol..

